# Sonidegib for the Treatment of Advanced Basal Cell Carcinoma

**DOI:** 10.3389/fonc.2020.582866

**Published:** 2020-10-30

**Authors:** Gabriella Brancaccio, Federico Pea, Elvira Moscarella, Giuseppe Argenziano

**Affiliations:** ^1^ Dermatology Unit, University of Campania “Luigi Vanvitelli”, Naples, Italy; ^2^ Department of Medicine, University of Udine, Udine, Italy; ^3^ Institute of Clinical Pharmacology, Azienda Ospedaliero-Universitaria Santa Maria Della Misericordia, Azienda Sanitaria Universitaria Friuli Centrale, Udine, Italy

**Keywords:** basal cell carcinoma, advanced basal cell carcinoma, hedgehog inhibitors, sonidegib, skin cancer

## Abstract

Basal cell carcinoma (BCC) accounts for almost 80% of skin cancers, and its healthcare workload burden is substantial within dermatology departments. Although most BCCs are small, well-defined tumors amenable of surgery or conservative procedures, in a small proportion of patients, BCCs can progress to an advanced stage including locally advanced BCC. The goal of the clinician in the treatment of BCC should be the right therapeutic approach at diagnosis, and different guidelines propose treatment strategies in order to prevent relapses or disease progression. In case of unresectable and untreatable BCC with radiotherapy, the first-choice medical therapy is Hedgehog-GLI (HH) pathway inhibitors. Sonidegib was approved by the U.S. Food and Drug Administration (FDA) and European Medicines Agency (EMA) as a first-line treatment for adult patients with locally advanced BCC, becoming the second HH pathway inhibitor receiving approval after vismodegib. In this review, data on pharmacology, safety, tolerability, and efficacy of sonidegib are summarized and compared to those of vismodegib. Lastly, indications on the management of advanced basal cell carcinoma based on author’s clinical experience are provided.

## Introduction

Basal cell carcinoma (BCC) accounts for almost 80% of skin cancers, and its oncogenesis rely on the interplay between constitutional predisposition (genotypic and phenotypic characteristics) and subsequent exposure to environmental risk factors, with ultraviolet radiation exposure as the principal one (1).

Actual BCC tumor burden is much greater in the population than it is apparent from normal incidence rates. Many reasons make the true BCC incidence difficult to calculate as 1) routine recording of BCC is often not performed by cancer registries; 2) in clinical practice not all the BCCs are histologically confirmed and 3) when recorded, often only the first histologically confirmed BCC per patient is taken into account. These factors translate into a complete absence of BCC rates in the most accounted statistical datasets (2), where it is even excluded from the group of non-melanoma skin cancer. However, the healthcare workload burden and cost of BCC are substantial within dermatology departments (3), and it is even much higher considering the subset of advanced BCC which accounts for the highest morbidity due to cosmetic disfigurement and functional morbidity.

**Figure 1:**
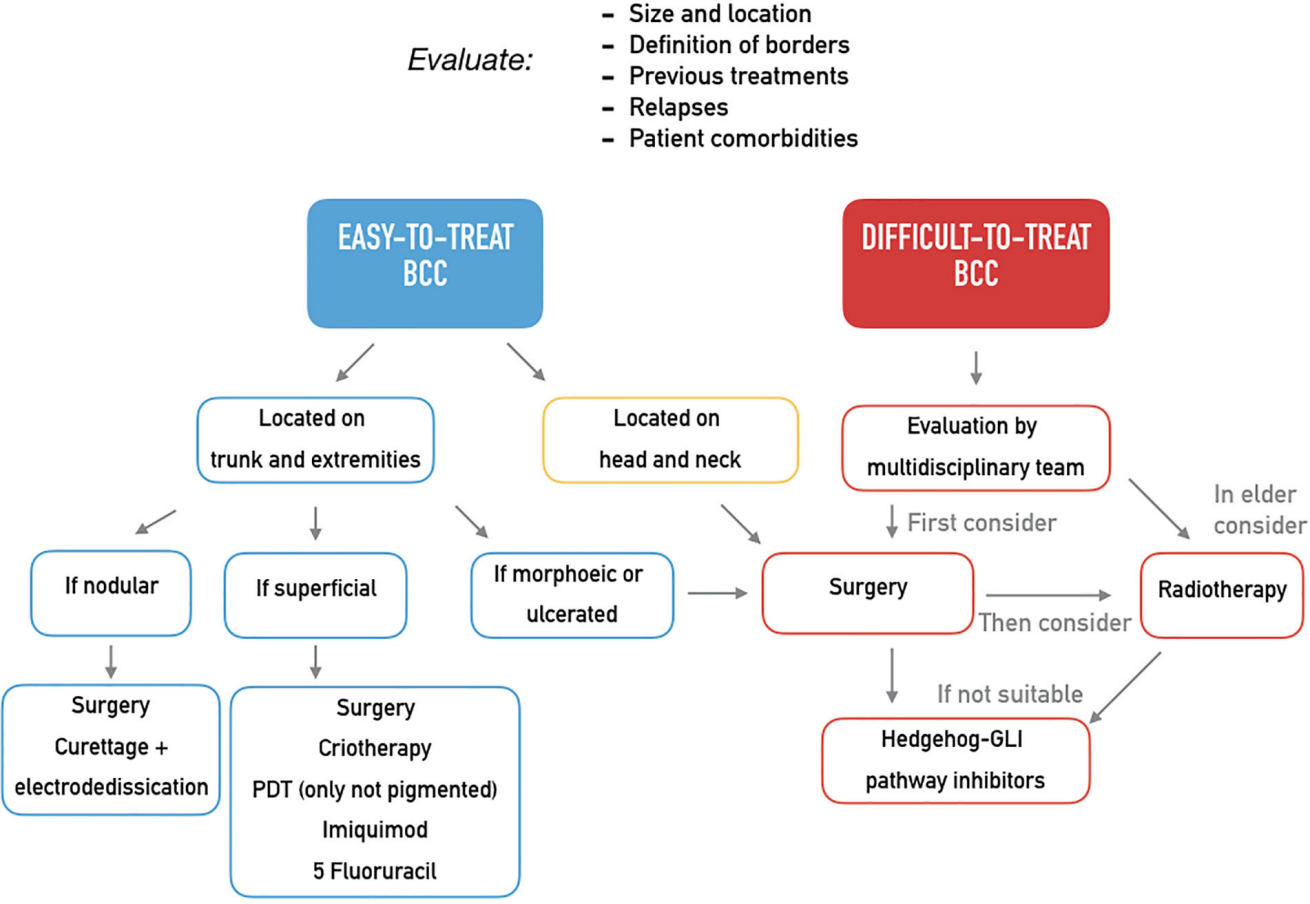
Expert opinion on the treatment of easy-to-treat and difficult-to-treat basal cell carcinomas.

Although most BCCs are small or intermediate-size, well-defined tumors amenable of surgery or conservative procedures, in a small proportion of patients, BCCs can progress to an advanced stage including metastatic BCC (mBCC) or locally advanced BCC (laBCC) (4). Advanced BCC is an entity not yet clearly defined as there is a lack of consensus on the diagnostic criteria which are hardly objectified. Usually, advanced BCCs are extended tumors characterized by destructive growth after multiple relapse, often located on the head and neck areas that have become difficult to treat through standard surgery and radiotherapy. In order to distinguish between BCCs that may progress to mBCC or laBCC, an innovative classification in easy-to-treat and difficult-to-treat BCCs has been recently proposed. It takes into account size, location, definition of borders, previous treatments, and related recurrences and even some patient’s characteristics as comorbidities interfering with surgery or reluctance to proposed treatments (5). The distinction between easy- and difficult-to-treat BCC may have practical implication considering the wide availability of therapeutic option for the first group of tumors and the need of an immediate resolutive treatment for the latter one (**Table 1**, **Figure 1**). Different guidelines (5, 6) propose treatment strategies in order to identify the better care pathway and, thus, prevent relapses or persistence of the tumor. The multidisciplinary approach is the mainstay of management of difficult**-**to**-**treat BCCs, that should be managed in a tertiary care center (referral center).

**Table 1:** Recommended therapeutic approach to easy-to-treat and difficult-to-treat BCCs (5).

	Treatment	Type of recommendation	Grade of recommendation–Level of evidence
Easy-to-treat BCC	Surgery	Highly effective in any type of BCC	A-3
	5% Imiquimod (sBCC)	Effective in sBCC	A-2
		Potential role in nBCC	B-2
	5% 5-Fluoruracil	Effective in sBCC	A-2
	Curettage + electrodedissication and cryoterapy	Potential role in low-risk BCC on the trunk and extremities	B-3
	PDT with MAL or ALA	Effective in sBCC and thin nBCC	A-1
Difficult-to-treat BCC	Surgery	Evaluation of suitability by multidisciplinary team	Expert opinion
	Radiotherapy	Role in elderly patients and patients not candidates for surgery (any BCC)	A-1
	HH inhibitors	To be offered in laBCC and mBCC	B-3

Surgery should be always considered as primary therapeutic option, even after neoadjuvant approaches. Mohs surgery should be performed in case of large, high-risk tumors located on the face, in case of surgery after a previous relapse, or in case of BCCs arising on a previous irradiated area, scars or areas of chronic inflammation. However, despite very high cure rate, Mohs surgery is a costly, time-consuming procedure that requires specialized training and has very little spread in some countries.

Radiotherapy should be taken into account as second-line treatment in elderly patients (>60 years old) suffering from a BCC not amenable of surgery. Radiotherapy is also an option in adjuvant setting in case of positive margins after primary excision. However, due to concerns with long-term sequelae as well as adverse events with intermediate onset, indication to radiotherapy may be questioned by the multidisciplinary team.

Once evolved to laBCC or mBCC, the most appropriate therapeutic option is the target therapy through Hedgehog inhibitors.

## Hedgehog-GLI Pathway and its Inhibitors

Hedgehog-GLI (HH) signaling plays a major role during the development and is involved in cell proliferation and differentiation (7, 8). The HH pathway is normally silenced in most adult tissues, and it was shown that it may be aberrantly activated in the pathogenesis of various types of tumors (9). This may promote the subsequent activation of transcription factors of the Glioma-associated oncogene (GLI) family, which may favor tumor proliferation (9). Smoothened (SMO) is the main transducer of HH signaling, and in the last few years, it has emerged as a promising therapeutic target for anticancer therapy. Natural and synthetic antagonists have been developed for SMO, and many have undergone clinical trials with varying degrees of success. SMO inhibition was first characterized through binding studies of cyclopamine, a natural steroidal alkaloid derived from *Veratrum californicum*. Derivatives of cyclopamine have been developed with the aim of increasing specificity and pharmacological potency while limiting side effects (10). The first HH pathway inhibitor to be approved by the FDA and EMA was vismodegib, a second-generation cyclopamine derivative. Later, sonidegib was approved by the FDA and EMA as a first-line treatment for adult patients with locally advanced BCC, becoming the second HH pathway inhibitor receiving approval (10). A new SMO inhibitor is also in development for topical administration in patients affected by Gorlin syndrome (11).

## Sonidegib for the Treatment of Advanced Basal Cell Carcinoma

Sonidegib is an oral small molecule that acts as a selective antagonist of the SMO receptor, a G protein-coupled receptor-like structure that is fundamental for the correct action of the HH signaling pathway (12).

Sonidegib exhibited dose- and exposure-dependent inhibition of the expression of the GLI homolog 1 in tumor and normal skin biopsies (13) and is currently indicated for the treatment of adults with advanced basal cell carcinomas at the daily dosage of 200 mg (12).

## Pharmacokinetic Profile

Sonidegib pharmacokinetics (PK) was studied in patients with cancer after a single dose ranging between 100 mg and 3000 mg (13). Under fasting condition, absorption resulted quite rapidly with a time to peak concentration (T_max_) of 2–4 h. Oral bioavailability (F_OS_) was quite low under fasted state as it was estimated to be around 6–7% after a single 800 mg dose in healthy volunteers (14). F_OS_ increased by 7.8-fold when in the presence of high-fat meal with an almost proportional increase in drug exposure of 7.4-fold in terms of area under the plasma concentration–time curve (AUC) from zero to infinity (15). For this reason, it is recommended that sonidegib is taken under fasting conditions, at least 1–2 h before meal (15).

One of the most interesting pharmacokinetic properties of sonidegib is represented by the wide distribution within tissues (14). A population pharmacokinetic analysis carried out among 351 patients who received sonidegib at a dose ranging between 100 mg and 3,000 mg showed that the volume of distribution (Vd) was of 9,170 L (15). This may explain why sonidegib may either achieve skin concentration sixfold higher than in plasma (15) or effectively cross the blood brain barrier (16). Sonidegib is bound for >97% to plasma protein in a concentration independent mode (15–17).

Sonidegib has a very long-elimination half-life of around 28 days (16, 18). This means that steady-state is reached after more or less 4 months from starting daily dosing treatment (16, 18), with an estimated accumulation of around 19-fold (13, 15). Sonidegib undergoes metabolism mainly *via* oxidation and hydrolysis by the 3A4 isoform of the cytochrome (CYP) P450 (15, 19). All of the metabolites are several-folds less pharmacologically active than the parent compound. Sonidegib is the main circulating moiety in plasma (36%), and both the parent compound and its metabolites are eliminated by the feces (overall 93% of the administered dose) (14).

Overall, the PK profile of sonidegib is quite different from that of the other SMO antagonist vismodegib (**Table 2**). Both drugs are very highly bound to plasma proteins (>97%), but the binding is concentration-independent for sonidegib (16, 17) and concentration-dependent for vismodegib (21, 22). The Vd is much higher for sonidegib than for vismodegib, accordingly to a major grade of lipophilicity. This may reflect in extensive accumulation of sonidegib within tissues, as documented by the finding of concentrations sixfold higher in the skin compared with plasma (15). Conversely, the distribution of vismodegib is mainly limited to the plasma and to the extracellular spaces (23). Theoretically, these differences in the distribution pattern might translate into potential differences in the pharmacodynamic profile of efficacy and toxicity of these two SMO inhibitors (20). Another relevant PK difference is related to the elimination half-life, which is three to fourfold longer for sonidegib (28–30 days) (16, 18) compared with vismodegib (4–12 days) (23, 24). This means that the time needed to achieve steady concentrations during continued treatment (namely the steady-state) is of around 3–4 months for sonidegib (16, 18) and of around 7–21 days for vismodegib (23, 24). The differences in time to steady state between the two HH inhibitors do not seem to correlate with the time to response, as the median time to response was 3.9 months for sonidegib in BOLT and 5.6 months for vismodegib in ERIVANCE trial (20).

**Table 2:** Comparative PK characteristic and efficacy of sonidegib and vismodegib.

PK	Sonidegib 200 mg daily	Vismodegib 150 mg daily
Plasma protein binding	>97% (concentration-independent) (8, 9),	>99% (concentration-dependent) (12, 13),
Vd (L)	9166 (7, 8),	16.4–26.6 (14)
t_1/2_ (days)	28–30 (8, 10),	4–12 (14, 16),
Time to steady-state (days)	90–120 (8, 10),	17–21 (14, 16),
Efficacy	Central review RECIST-like 18-month follow-up (BOLT trial) (20)	Central review RECIST 21-month follow-up (Erivance trial) (20)
Overall response rate n (%); 95% CI	40 (60.6); 47.8–72.4	30 (47.6); 35.5–60.6
Complete response n (%)	14 (21.2%)	14 (22.2%)
Partial response n (%)	26 (39.4%)	16 (25.4%)
Stable disease n (%)	20 (30.3%)	22 (34.9%)
Progressive disease n (%)	1 (1.5%)	8 (12.7%)
Unknown n (%)	5 (7.6%)	3 (4.8%)

RECIST, Response Evaluation Criteria in Solid Tumors. Adapted by Dummer et al. J Eur Acad Dermatol Venereol. 2020.

### Drug–Drug Interactions

Sonidegib is a substrate of CYP3A4 and it is expected that its pharmacokinetic profile may be altered by modulators of the activity of this metabolizing enzyme (15, 19). Thus, the recommendation on the EMA product label is to avoid co-administration with strong CYP 3A4 inhibitors or to reduce sonidegib dose to 200 mg every other day during co-treatment with strong CYP 3A4 inhibitors in order not to exceed a twofold increase in sonidegib exposure (15, 19). Similarly, co-treatment with strong CYP 3A4 inducers should be avoided (15, 19). However, if co-treatment with inducers is needed, sonidegib dose may be increased to 400–800 mg in order to prevent >80% reduction in sonidegib exposure (15, 19). Concomitant treatment with strong CYP inducers should be avoided in the case of vismodegib as well. The product label does not provide any advice on dose adjustment if co-administration is necessary [Erivedge EMA label].

### Pharmacokinetic Profile in Special Patient Populations

The pharmacokinetic behavior of sonidegib was evaluated also in special patient populations. The effect of mild to severe hepatic impairment on the pharmacokinetics of sonidegib was assessed in a phase 1 multicenter, open label, parallel-group study (25) concluding that in patients with any grade of hepatic impairment dose adjustments are unnecessary.

Sonidegib has not been studied in a dedicated pharmacokinetic study in patients with renal impairment. Based on the available data, sonidegib elimination *via* the kidney is negligible. A population pharmacokinetic analysis found that mild or moderate renal impairment did not have a significant effect on the apparent clearance of sonidegib, suggesting that dose adjustment is not necessary in patients with renal impairment. No efficacy and safety data are available in patients with severe renal impairment [Odomzo EMA label].

Additionally, safety and efficacy data in patients aged 65 years and older do not suggest that a dosage adjustment is required in these patients [Odomzo EMA label].

A population pharmacokinetic analysis of sonidegib was carried out among healthy volunteers and patients with advanced solid tumors (18). Covariate analysis showed that age, weight, gender, ethnicity, mild hepatic impairment, mild and moderate renal impairment did not affect sonidegib pharmacokinetics. This means that no sonidegib dose adjustment is indicated in relation to these conditions. Conversely, clinically relevant effects on sonidegib F_OS_ were induced by high-fat meal (fivefold increase), and by co-administration of proton pump inhibitors (30% decrease). In regard to the former effect, it is recommended that sonidegib is assumed under fasted condition for avoiding unpredictable overexposure (15). In regard to the latter effect, a phase 1 study carried out among 42 healthy volunteers showed that co-administration of esomeprazole (40 mg 5-days pretreatment plus combination on day 6) with a single 200 mg dose of sonidegib resulted in a modest reduction of sonidegib absorption under fasted conditions (decreased sonidegib AUC by 32-38%) (26).

## Tolerability and Safety

The safety and the tolerability of sonidegib was assessed in the double-blind, phase 2 pivotal trial (BOLT) in which patients with locally advanced or metastatic basal cell carcinoma were randomized to receive 200 or 800 mg oral sonidegib daily (27).

A comprehensive analysis assessed whether an exposure–response relationship would exist for effectiveness and safety of sonidegib among patients with advanced solid tumors (28). For the exposure–efficacy analysis, data from 190 patients receiving sonidegib at 200 or 800 mg daily were included. Logistic regression analysis showed no relationship between sonidegib exposure in terms of trough level (C_min_) resulting from 200 or 800 mg doses at week 5 and the objective response rate in terms of complete and/or partial response. Exposure–safety analysis was carried out among 336 patients receiving dosages ranging from 100 to 3,000 mg once daily and 250 to 750 mg twice daily. The findings showed that increased exposure was associated with a greater risk of grades 3–4 creatine kinase (CK) elevation, and that the risk was lower in females *vs.* males. Consistently, it is recommended that CK level is monitored periodically throughout the duration of treatment with sonidegib (29).

A pooled analysis of the change in the QT interval was carried out for assessing the eventual prolongation QT caused by sonidegib. Data coming from four patient studies (n = 341) were merged with those coming from four healthy volunteer studies (n = 204) (30). Overall, data showed that sonidegib did not cause QTc prolongation as ΔQTc were always <5 ms both for the 200 and 800 mg dose.

With regard to tolerability, the most frequent adverse events (AEs) resulted in muscle spasms, alopecia, and dysgeusia, mostly of grade 1–2 (17). The most common grade 3–4 AEs occurring in ≥2% of patients receiving the 200 mg daily dose were fatigue, weight decrease, and muscle spasms. Even if data from the two pivotal studies are not directly comparable, sonidegib resulted in being associated with the same AEs of vismodegib but with an approximately 10% lower incidence (4). AEs reported with sonidegib were also slightly less severe and with a slightly longer median time to onset (4). Specifically, the median time to onset of the most frequent AEs with vismodegib 150 mg and sonidegib 200 mg, namely muscle spasms, alopecia and dysgeusia, were 1.89 *vs* 2.07 months, 3.38 *vs* 5.55 months and 1.48 *vs* 3.71 months, respectively.

## Efficacy

The phase 2 trial (BOLT) that led to the approval in both US and Europe compared sonidegib at a dosage of 200 and 800 mg in patients affected by laBCC (n = 194) and mBCC (n = 36). As sonidegib 200 mg demonstrated a better benefit-risk profile than sonidegib 800 mg, we will focus only on the former, which is the approved dose in the setting of laBCC (15).

Primary endpoint of the BOLT trial was overall response rate (ORR) by central review, while secondary endpoints were ORR by investigator review, duration of response (DOR), progression free survival (PFS), overall survival (OS), time to response, safety and quality of life (QoL). Noteworthy, assessment of laBCC in BOLT trial was performed using the BCC-modified RECIST criteria (mRECIST) (27). BCC-mRECIST is a multimodal assessment method integrating magnetic resonance imaging per RECISTv1.1, standard and annotated color photography per WHO guidelines, and histology in multiple biopsy specimens surveying the lesion area. Overall, these criteria for assessing partial and complete response, as well as progression disease, are more stringent compared to the RECISTv1.1 criteria used in vismodegib studies (4). mRECIST is more likely to detect minimal signs of disease and disease progression, thus classifying a given treatment response as partial, whereas the same response may be considered as complete using RECIST. Similarly, mRECIST is more likely to detect signs of slight disease progression that may be classified as stable disease (SD) under RECIST (20). This aspect is crucial when comparing efficacy data from sonidegib and vismodegib trial analyses (17, 27, 31, 32) (**Table 2**). Despite similar baseline patient characteristics, endpoints, and role of central and investigator review, the difference in assessment criteria makes a head-to-head comparison of the two drugs difficult. However, in the 30-month analysis of the BOLT study, a pre-planned analysis adjusted the outcomes from BOLT with RECIST-like criteria. As underlined in a recent expert opinion paper, the most correct match is between adjusted ORR of sonidegib and ORR of vismodegib at the closest follow-up time points across the studies with central review (20). At 21-month follow-up, vismodegib ORR was 47.6%, with 22.2% complete response (CR) and 25.4% partial response (PR). At 18-month follow-up, adjusted ORR of sonidegib was 60.6% with 21.2% CR and 39.4% PR. Adjusting efficacy data using RECIST criteria make just a slight increase in sonidegib overall response rate (ORR) (from 56.1 to 60.6%) while the number of CR increases significantly at the expense of PR. The rate of progressive disease (PD) is higher for vismodegib than for sonidegib (12.7 and 1.5%, respectively) (20), and this data is consistent with reports of acquired resistance during treatment with vismodegib (4). However, it is likely that the responsible genomic mutations affecting SMO confer resistance to different SMO inhibitors. Further studies are needed to find the right therapeutic strategy in constitutionally or acquired resistant laBCC, through drug associations or different molecules. Lastly, the centrally reviewed median duration of response (mDOR) and median progression free survival (mPFS) with sonidegib at 30 months were longer than vismodegib at 21 months (17, 31). The longest (39 months) follow-up report of vismodegib includes only investigator reviewed data, therefore is not appropriate for a comparison (32). However, the investigator reviewed mDOR results are longer with vismodegib.

## Clinical Implications and Conclusions

The goal of the clinician in the treatment of BCC should be the right therapeutic approach at diagnosis, thus preventing the evolution into laBCC or mBCC. Many treatments are available depending on the clinical features of the primitive lesion and on patient characteristics (**Table 1**), and the distinction into easy-to-treat and difficult-to-treat BCCs may be helpful in the clinical practice (**Figure 1**). Easy-to-treat BCCs may be properly managed by the territorial health care or in the private practice, while difficult-to-treat BCCs should be referred to a secondary/tertiary care center in order to be evaluated by a multidisciplinary team. Obviously, the experience of each center differs from one country to another and in the same country and may influence the therapeutic decision, but general recommendations should be followed (5).

For the treatment of difficult-to-treat BCCs, surgery should be the first therapeutic option, but it should be carefully planned, and appropriate imaging to determine the extent of the tumor should be performed when perineural involvement or bone invasion is suspected. When available, Mohs surgery should be preferred. Radiotherapy is an alternative option in elderly patient affected by BCCs not amenable of surgery or in patients who are not candidates to surgery; it is devoted to elderly people because the potential risk of very-long-term trophic disorders is not well addressed (5).

In case of unresectable and untreatable BCC with radiotherapy (laBCC), the first-choice medical therapy is HH pathway inhibitors. Chemotherapy showed a low response rate and a short duration of response in few reports, so it can be considered a last-line treatment, while studies on the efficacy of immunotherapy in BCC are currently ongoing (5).

To date, the choice between the two HH inhibitors available, vismodegib and sonidegib, is based on expert opinion and indirect comparison, as a head-to-head trial is not available. However, a subset of patients who could benefit more from one drug than another has not been clearly identified. Vismodegib, being the first approved HH inhibitor, has been used for longer time and real-world data are available. Although no laboratory tests are required by label (except for pregnancy test), we routinely perform a metabolic panel every 1–2 months, depending on patient comorbidities, with special attention to liver and kidney functionality and creatinine kinase levels. We experienced the efficacy of vismodegib in many laBCC patients, with both complete and partial responses, but also some disease progressions after the onset of resistance, as reported in literature. The main pitfall is the adherence to a long, otherwise chronic, treatment due to the onset of adverse events and their impact on quality of life. The most reported and least tolerated side effect seems to be muscle spasms; it occurs relatively early during the treatment and implementation through magnesium or levocarnitine shows a mild effectiveness in few cases. Dysgeusia and alopecia are of later onset but equally impairing AEs. To overcome this issue, different preventive and management strategies have been proposed, mainly drug holydays. However, since no dose adjustments are present in the vismodegib data sheet, any individual modifications that may be introduced are off-label.

Sonidegib is the latest HH inhibitor to be approved; thus its real-life experience is being built. However, both trial results and clinical experience confirm a similar efficacy profile to vismodegib. Comparing the adjusted results of BOLT trial at 18-month follow-up to the results of ERIVANCE trial at 21-month follow-up points out slightly higher ORR and PR, similar CR and SD, and a lower PD for sonidegib (20). Like vismodegib, also sonidegib is not contraindicated in any specific patient subset, but monitoring of CK levels is indicated. We usually prescribe the same laboratory tests for vismodegib. With regard to tolerability, sonidegib shares the same class-dependent AEs of vismodegib; however, they seem to be less frequent and with a slightly longer time to onset, probably due to a different pharmacokinetic profile. The availability of an alternative administration schedule included in the label (200 mg every other day) is very helpful in managing the entity of specific AEs, such as high CK levels, and thus the rate of treatment discontinuation may be lowered.

To understand which patient could benefit from vismodegib or sonidegib, real-world data on the latter drug are needed. Only one case report described the experience of a laBCC successfully treated with sonidegib with complete response and with no side effects (33). A case series collecting experience in our center is under review. However, making any definitive directives for the choice between the two HHi is premature. Besides real-world data on sonidegib use, a head-to-head trial should be designed in order to produce more reliable comparative data. Also, intermittent trials, sequential trials, or cross-over trials of the two HH inhibitors in laBCC patients who discontinued treatment due to AEs may demonstrate the impact of the pharmacokinetic profile differences and improve the awareness of the clinician on the use of HH inhibitors.

## Author Contributions

All the authors contributed equally to this work. All authors contributed to the article and approved the submitted version.

## Funding

This study received an unrestricted grant from SunPharma.

## Conflict of Interest

The authors declare that this study received funding from Sun Pharma. The funder was not involved in the study design, collection, analysis, interpretation of data, the writing of this article, or the decision to submit it for publication.
